# Establishment and characterization of patient-derived head and neck cancer models from surgical specimens and endoscopic biopsies

**DOI:** 10.1186/s13046-021-02047-w

**Published:** 2021-08-06

**Authors:** Daniel Strüder, Theresa Momper, Nina Irmscher, Mareike Krause, Jan Liese, Sebastian Schraven, Annette Zimpfer, Sarah Zonnur, Ann-Sophie Burmeister, Björn Schneider, Bernhard Frerich, Robert Mlynski, Christina Große-Thie, Christian Junghanss, Claudia Maletzki

**Affiliations:** 1 Department of Otorhinolaryngology, Head and Neck Surgery “Otto Koerner”, Rostock University Medical Center, Rostock, Germany; 2 Department of Internal Medicine, Medical Clinic III - Hematology, Oncology, Palliative Medicine, Rostock University Medical Center, Schillingallee 70, 18057 Rostock, Germany; 3 Department of Oral and Maxillofacial Surgery, Facial Plastic Surgery, Rostock University Medical Center, Rostock, Germany; 4 Institute of Pathology, Rostock University Medical Center, Rostock, Germany

**Keywords:** Primary cancer, HPV positive and negative, Individual tumor models, Immune cell infiltration, Recurrence, Metastasis, Non-resectable, Endoscopic biopsy, Head and neck squamous cell carcinoma

## Abstract

**Background:**

Head and neck squamous cell carcinoma (HNSCC) is heterogeneous in etiology, phenotype and biology. Patient-derived xenografts (PDX) maintain morphology and molecular profiling of the original tumors and have become a standard “Avatar” model for human cancer research. However, restricted availability of tumor samples hindered the widespread use of PDX. Most PDX-projects include only surgical specimens because reliable engraftment from biopsies is missing. Therefore, sample collection is limited and excludes recurrent and metastatic, non-resectable cancer from preclinical models as well as future personalized medicine.

**Methods:**

This study compares the PDX-take rate, -growth, histopathology, and molecular characteristics of endoscopic specimens with surgical specimens. HNSCC samples (*n* = 55) were collected ad hoc, fresh frozen and implanted into NOD.Cg-Prkdc^scid^Il2rg^tm1Wjl^/SzJ mice.

**Results:**

Engraftment was successful in both sample types. However, engraftment rate was lower (21 *vs.* 52%) and growth delayed (11.2 *vs.* 6.7 weeks) for endoscopic biopsies. Following engraftment, growth kinetic was similar. Comparisons of primary tumors and corresponding PDX models confirmed preservation of histomorphology (HE histology) and molecular profile (Illumina Cancer Hotspot Panel) of the patients’ tumors. Accompanying flow cytometry on primary tumor specimens revealed a heterogeneous tumor microenvironment among individual cases and identified M2-like macrophages as positive predictors for engraftment. Vice versa, a high PD-L1 expression (combined positive score on tumor/immune cells) predicted PDX rejection.

**Conclusion:**

Including biopsy samples from locally advanced or metastatic lesions from patients with non-surgical treatment strategies, increases the availability of PDX for basic and translational research. This facilitates (pre-) clinical studies for individual response prediction based on immunological biomarkers.

**Supplementary Information:**

The online version contains supplementary material available at 10.1186/s13046-021-02047-w.

## Background

Head and neck squamous cell cancer (HNSCC) is the 7^th^ most common cancer worldwide and associated with a poor outcome [1–5]. Despite aggressive surgery, radiation- and chemotherapy, ~ 50% of patients die, while survivors suffer from pain, dysphagia and dysphonia [6]. Today, recurrence, and treatment response are difficult to predict, because of inter- and intratumoral heterogeneity in etiology, phenotype, and biology. Preclinical models must represent this heterogeneity to identify predictive biomarkers and develop effective personalized medicine.

Patient-derived xenografts (PDX), generated by implantation of human cancer tissue into immunodeficient mice, are considered as the gold standard for preclinical cancer research [7–11]. In early passages, PDXs faithfully recapitulate the original tumors’ cellular, molecular and histopathological structures as well as drug response and clinical outcome [8, 12]. Thus, PDX provide an excellent platform for translational research on biomarkers and drug development (including setup of clinical trials) [12–15].

However, the restricted availability of tumor samples hinders widespread use of PDX in HNSCC. The PDX take rate for surgical HNSCC-specimens (50–75%) is comparable to other solid tumors (lung ~ 30–60%, CRC 70%, pancreas ~ 50%) [14–21], but the availability of suitable specimens is limited. In early stage tumors, the pathologist needs most of the surgery specimen for tumor staging and margin controls, while advanced (metastatic) disease is mostly treated with chemo-, immune- or radiation therapy (without surgery). Therefore, PDX from surgery specimens exclude recurrent and advanced (metastatic) HNSCC from preclinical models and personalized medicine.

In lung-, gastrointestinal-, pancreatic-, bladder- and skin cancer PDX-projects, the lack of surgical specimens has led to the use of endoscopic- and needle biopsies [15, 22, 23]. Engraftment rates were lower for biopsies (33–60%) *vs*. surgery specimens (40–100%), but PDX formation from minimally invasive procedures was possible. For HNSCC, endoscopic PDX sampling appears technically suitable, because of exophytic tumor growth and diagnostic sampling from both the vital margin and the necrotic center of the lesion. Two recent HNSCC studies regularly used biopsies for PDX formation [24, 25]. Lilja-Fischer et al*.* reported a biopsy take rate of 33% in oropharyngeal cancer [24] while Kang et al*.* observed 100% engraftment efficacy for biopsies in a small and defined patient cohort [26]. Therefore, HNSCC PDX biopsy engraftment appears to be feasible. However, take rate, time to engraftment and contributing factors of endoscopic biopsies for HNSCC PDX formation remain unclear.

In this study, we describe a practical and straightforward method to establish p16 positive and negative PDX models using endoscopic and surgical tumor samples. Performing a side-by-side comparison, we show that the engraftment rate was lower for biopsies, but PDX had similar growth kinetics once established. With this setup, a PDX library was created including clinical characteristics, pathological analysis as well as molecular and immunological data of the tumor microenvironment.

## Materials and methods

### Tumor sample preparation

HNSCC samples were donated from consecutive patients undergoing an endoscopic biopsy or surgery at a University Medical Center from 06/2018 through 01/2020. Patients with pathologically proven or suspicion of HNSCC and the following characteristics were eligible: 1) HNSCC of the oral cavity, oropharynx, hypopharynx, larynx and neck lymph node metastases; 2) size of the tumor > 2 cm; 3) primary disease or recurrence; 4) > 18 years of age. Written informed consent was obtained according to the local Ethics Committee (reference number A2018-0003) and the guidelines for the use of human material. Blood sampling (2 × 7.5 ml Heparin) was performed prior to endoscopy/surgery. Endoscopic biopsies were sampled using endoscopic scissors as required for the routine diagnostic procedure. Surgery specimens were obtained by open (e.g. pharyngotomy, laryngectomy) or transoral approach (e.g. laser, radio frequency).

**Fig. 1:**
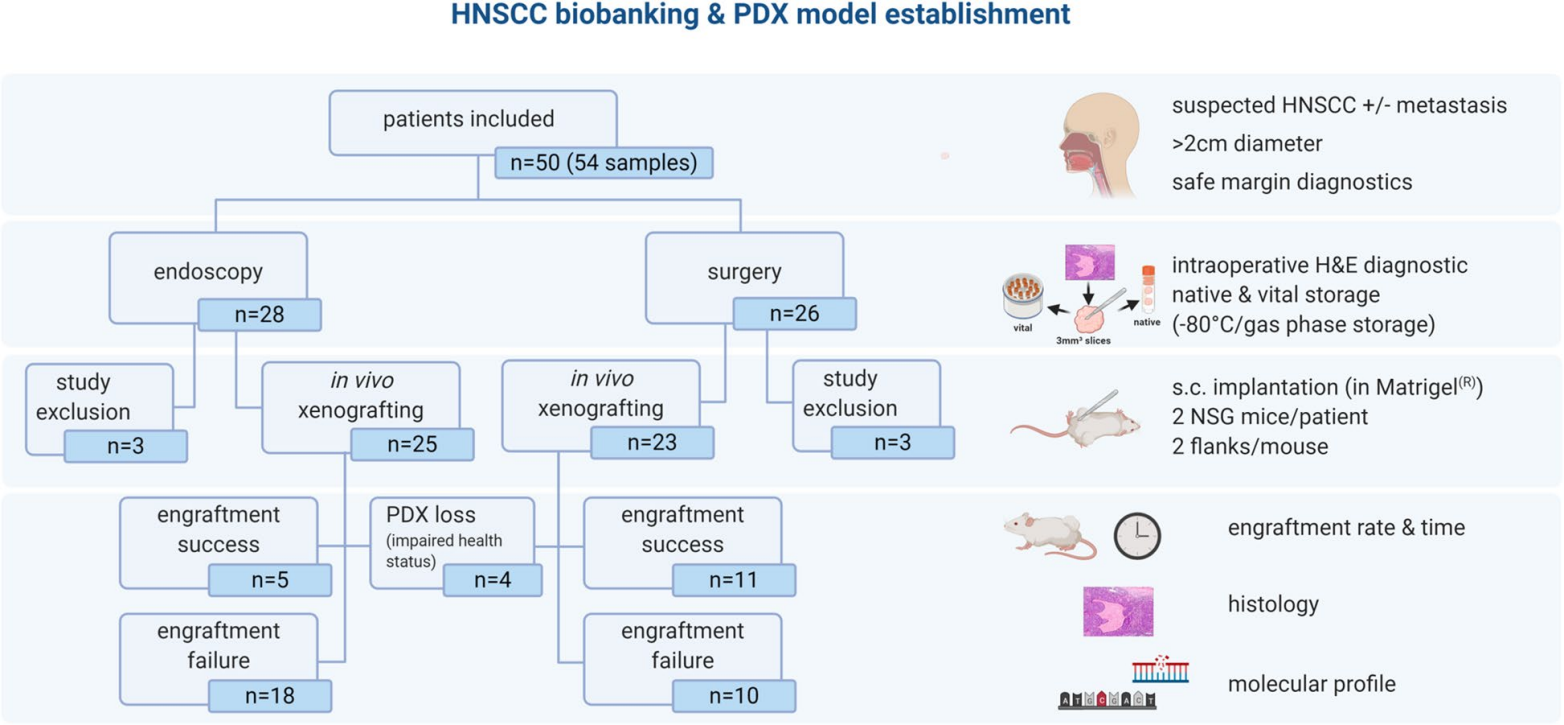
Workflow of sample processing, implantation and PDX validation. HNSCC patients that underwent diagnostic endoscopy or surgery were included in the study. Following resection/biopsy, the sample underwent H&E diagnostics for squamous cell carcinoma. The pathologist chose a part of viable cancer for fresh frozen fragments (within 120 min post removal). Next, thawed 3 × 3 × 3 mm^3^ fragments were implanted subcutaneously into NSG mice. Finally, the engraftment efficacy, histology and molecular pathology were analyzed. Created with *biorender.com*

Immediately after dissection, the samples were sent to the Institute of Pathology (in room temperature NaCl 0.9%) for instantaneous H&E section. The pathologist removed tumor tissue for routine diagnostics, necrotic/fibrotic areas and provided macroscopically vital tumor tissue for the experimental laboratory. All specimens of at least 5 × 5 × 5 mm^3^ were accepted and processed within 120 min of ischemia time using a clean bench. Tumor samples were cut into fragments of 3 × 3 × 3 mm^3^ and split: Two pieces were snap frozen and stored in liquid nitrogen for molecular analysis. The remaining fragments were frozen viable (FCS, 10% DMSO) and stored at -80 °C for xenografting (Fig. 1).

### Ethical statement

All animal experiments were approved by the local governmental authority (approval number: 7221.3‐1‐066/18), in accordance with the governmental animal protection law and the EU Guideline 2010/63/EU. For in vivo engraftment, six-week-old female NOD.Cg-Prkdc^scid^Il2rg^tm1^Wjl (NSG, Charles River Laboratories, Lyon, France) mice were used as recipients. Mice were bred in the local animal core facility under specific pathogen‐free conditions. During the experiment, mice were kept in type III cages (Zoonlab GmbH, Castrop‐Rauxel, Germany) at 12‐h dark:light cycle, the temperature of 21 ± 2 °C, and relative humidity of 60 ± 20% with food (pellets, 10 mm, ssniff‐Spezialdiäten GmbH, Soest, Germany) and tap water ad libitum. During their whole lifetime, all animals received enrichment as mouse-igloos (ANT Tierhaltungsbedarf, Buxtehude, Germany), nesting material (shredded tissue paper, Verbandmittel GmbH, Frankenberg, Deutschland, paper roles (75 × 38 mm, H 0528–151, ssniff‐Spezialdiäten GmbH), and wooden sticks (40 × 16 × 10 mm, Abedd, Vienna, Austria).

### PDX generation

After melting of the frozen samples, DMSO was removed and the tumor fragments were embedded in Matrigel (Corning® Matrigel® Basement Membrane Matrix, Wiesbaden, Germany) for 10 min. Meanwhile, the NSG-mice were anesthetized using Ketamin/Xylazin (dose: 90/6 mg/kg bw). After verification of sufficient anesthesia, both hind flanks were shaved, iodine disinfected and incised (3 mm). Tumor fragments were implanted subcutaneously and wounds were closed using simple interrupted sutures (Ethicon 6–0, Johnson & Johnson GmbH, Neuss, Germany) followed by iodine disinfection. Mice were placed under a heating lamp during recovery from anaesthesia and received analgesia (Metamizol 1250 mg/l, in drinking water) (pre- and) post-surgery to reduce pain.

After recovery, the flank tumors were measured weekly using a caliper. If no tumor growth occurred for 6 months, mice were euthanized. When flank tumors grew and reached a maximum size of 1.5 × 1.5 × 1.5 cm^3^, euthanasia and tissue collection was performed. The tumor was minced as described for the primary sample. Briefly, two fragments were used for molecular and histological analysis and the remaining fragments were viable frozen for further passaging. Tumor nomenclature was as followed: HNSCC [serial number] P [passage P0/P1] M [mouse M1/M2]. The patients’ serial numbers are maintained throughout all PDX passages.

### Histology and immunohistochemistry of patient tumor tissue and PDX

Morphology of patient tumor tissue and their corresponding PDX models was studied by an expert pathologist. Histopathology of primary tumors and PDX followed standard protocols for HNSCC staging including HE staining and immunohistochemistry; antibodies: anti-CD8 (Clone C8/144B, Dako, Hamburg, Germany, anti-p16 (clone: G175-405, BD Bioscience, Heidelberg, Germany), anti-Ki-67 (Clone Mib-1, Dako), anti-PD-L1 (Clone 22C3, Dako).

### Molecular pathology

Nucleic acids and proteins were isolated from snap frozen primary tumors and PDX [mean weight: 16.6 mg]. Simultaneous purification of DNA, RNA, and protein (from the same tissue sample) was performed using AllPrep DNA/RNA/Protein Mini Kit (Qiagen, Hilden, Germany) Isolation was executed according to the manufacturers’ instructions: (I) tissue disruption and homogenization; (II) RNA isolation; (III) protein isolation; and (IV) gDNA isolation. Isolated nucleic acids and proteins were stored at -80 °C. gDNA samples were used to detect genomic alterations using the Illumina Cancer Hotspot Panel (Illumina, Berlin, Germany) covering mutations in 50 different genes with an iSeq100 sequencing system (Illumina) according to the manufacturer’s protocols. For human papilloma virus (HPV) testing, a commercially available kit was used (VisionArray HPV Chip 1.0, ZytoVision, Bremerhaven, Germany) and applied according to the manufacturer’s instructions.

### Multi-color flow cytometry

Surface marker expression on single tumor cell suspensions was assessed by multi-color flow using a panel of human-specific conjugated antibodies (mAb, 1 μg each): anti-CD3 FITC (clone OKT-3), anti-CD4 PE (clone IT4), anti-CD8 PE (clone MEM-31), anti-CD56 PE (clone MEM-188), anti-CD16 APC (clone 3G8), anti-CD274 PECy7 (clone 29E.2A3), anti-CD70 FITC (clone 113–16), anti-CD14 FITC (clone 63D3), anti-CD204 PE (clone 7C9C20), anti-CD169 APC (clone 7–239), anti-CD163 PECy7 (clone GHI/61). Whole blood and tumor samples were stained for 30 min (4 °C). Afterwards, erythrocytes were lysed using 155 mM NH_4_Cl (MERCK Millipore, Darmstadt, Germany), 10 mM KHCO_3_ (MERCK Millipore), and 0.1 mM EDTA (Applichem, Darmstadt, Germany). Negative controls were stained with the appropriate isotypes (Biolegend) or left unstained. Cells were washed, resuspended in PBS and analyzed by flow cytometry on a FACSVerse Cytometer (BD Pharmingen). Data analysis was performed using BD FACSuite software (BD Pharmingen).

### Statistics

Statistical evaluation was performed using GraphPad PRISM software, version 5.02 (GraphPad, San Diego, USA). Values are reported as the mean ± SD. After proving the assumption of normality, differences between biopsies and surgery specimens were calculated using the unpaired Student’s t-test. If normality failed, the non-parametric Mann–Whitney *U* test was applied. Multiple comparisons were done using one way ANOVA on ranks (Bonferroni’s Multiple Comparison Test). Spearman nonparametric correlation was used to calculate correlations between individual parameters (two-tailed *P* value). The criterion for significance was taken to be *p* < 0.05.

## Results

### Patient and sample characteristics

**Fig. 2:**
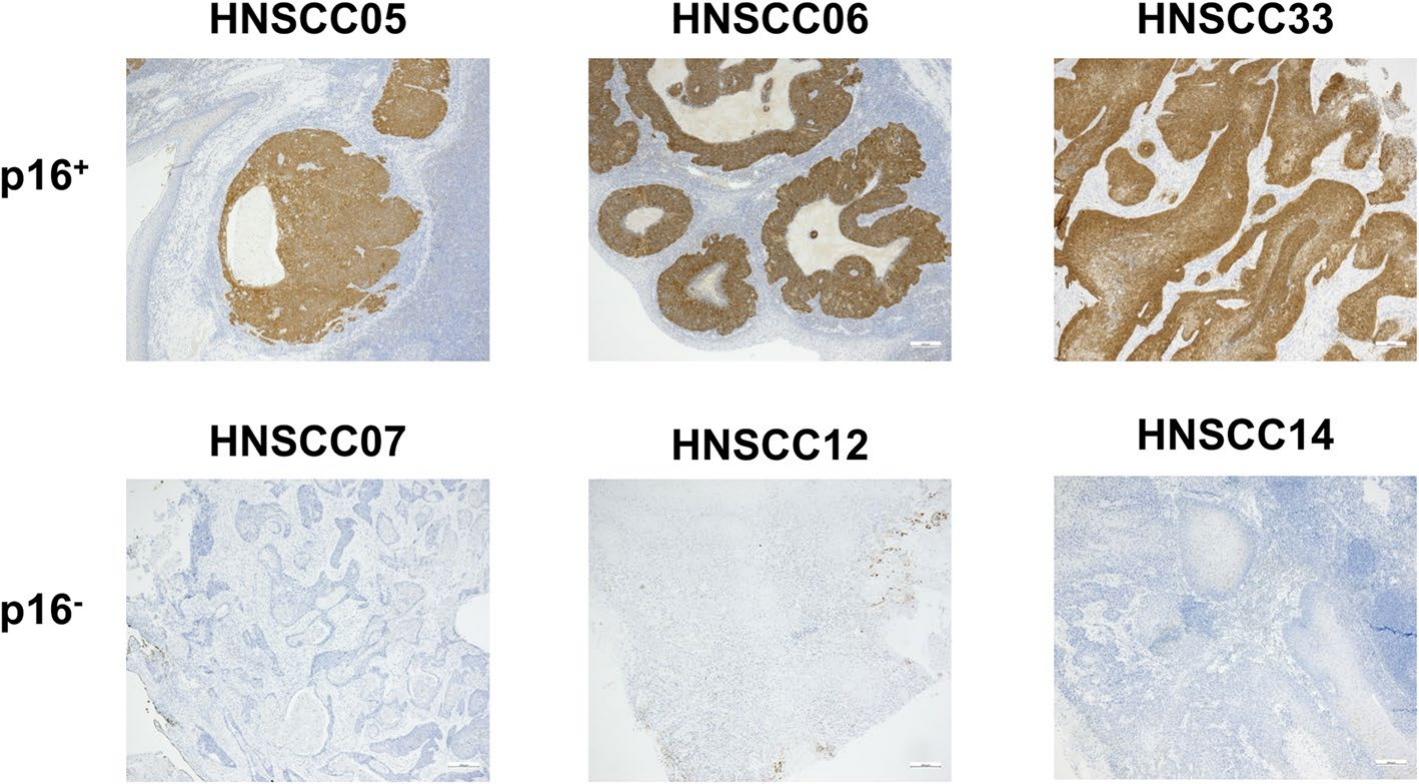
Immunohistochemistry of p16. Representative images of p16^+^ and p16^−^ cases are shown (5 × magnification). Immunohistochemistry was done as described in material & methods using clone: G175-405

Fifty patients were included (male:female: 41:9) with high performance status (ECOG 1.06 ± 0.88) with a median age of 64.0 years (range 46 to 82) (Table 1 and Fig. 1). Most patients were smokers (68%, ≥ 10 py), without critical alcohol consumption (54%). The majority of patients presented with locally advanced disease (T4a/4b) and four with a recurrence after (chemo)-radiation. Tumors were localized in the oropharynx (*n* = 19), oral cavity (*n* = 17), larynx (*n* = 11) and hypopharynx (*n* = 5) (Table 1). Neck metastases were collected from four patients (primary + related metastasis: HNSCC40/40II/40III and HNSCC45/46; metastasis only: HNSCC08 and HNSCC48). One case was a HNSCC lymph node metastasis of unknown primary (CUP) (HNSCC37). Thirteen of the tumors were p16^+^ (determined by p16 immunoreactivity, representative images of p16^+^ and p16^−^ cases are given in Fig. 2). To analyze whether p16 positivity is the result of HPV infection, molecular HPV testing was done (Supplementary Table 1). HPV was confirmed in 12 cases, whereas one case (HNSCC26) was finally classified as p16^+^/HPV^−^.

**Table 1:** Characteristics of the 50 study participants (Σ = 56 samples)

Group characteristics	Surgery Σ *n* = 26		Biopsy Σ *n* = 28	
	**%**	***n***	**%**	***n***
Female	15	4	18	5
Male	85	22	82	23
Age [years]	64.8	± 9.4	64.5	± 8.1
Performance status [ECOG]	1.0	± 0.7	1.1	± 1.0
**Noxae**				
Smoking [> 10 py]	73	19	68	19
Alcohol [> 1 drink/d]	58	15	32	9
**Localization**	38	10		
Oral cavity	23	6	25	7
Oropharnyx	12	3	46	13
Hypopharynx	27	7	7	2
Larynx			18	5
CUP			4	1
**p16 status**				
Positive	15	4	32	9
Negative	85	22	68	19
**G1/G2/G3** [%]	6/73/21		5/70/25	
**T1/T2/T3/T4** [%]	13/27/30/30		-/17/29/54*	
**N0/N1/N2/N3** [%]	56/30/5/9		15/35/60/0*	

Values depict absolute/relative numbers and mean ± SD. Chi-square test was performed to analyze homogeneity of surgery *vs*. biopsy groups (*p < 0.05)

*Abbreviations*: *py* pack years, *CUP* cancer of unknown primary, *G1/2/3* grading

The mean sample size of the tumor piece was 1.2 ± 1.6 cm^3^. In some cases of endoscopic biopsies, several pieces were obtained. Biopsy samples were smaller than surgical specimens (mean size: 0.9 cm^3^ *vs*. 1.3 cm^3^). The ischemia time was the same for biopsies and surgery samples (< 120 min, mean: 89.5 ± 33.6 min).

Detailed information on clinical follow-up, including adjuvant treatment, is summarized in Table 1. So far, four patients died because of progressive disease (HNSCC21, HNSCC31, HNSCC48, and HNSCC51).

### PDX engraftment rate of biopsies and surgical specimens

Fourty eight cryopreserved individual HNSCC tumors were implanted subcutaneously into NSG mice (Table 1). Six HNSCC cases did not undergo PDX engraftment because of limitations in tumor quantity/quality and unspecific tumor type (CUP). Additional four HNSCC cases were lost because of impaired health of laboratory animals. Finally, 44 cases were included in data acquisition: 23 biopsy samples and 21 surgery samples.

**Fig. 3:**
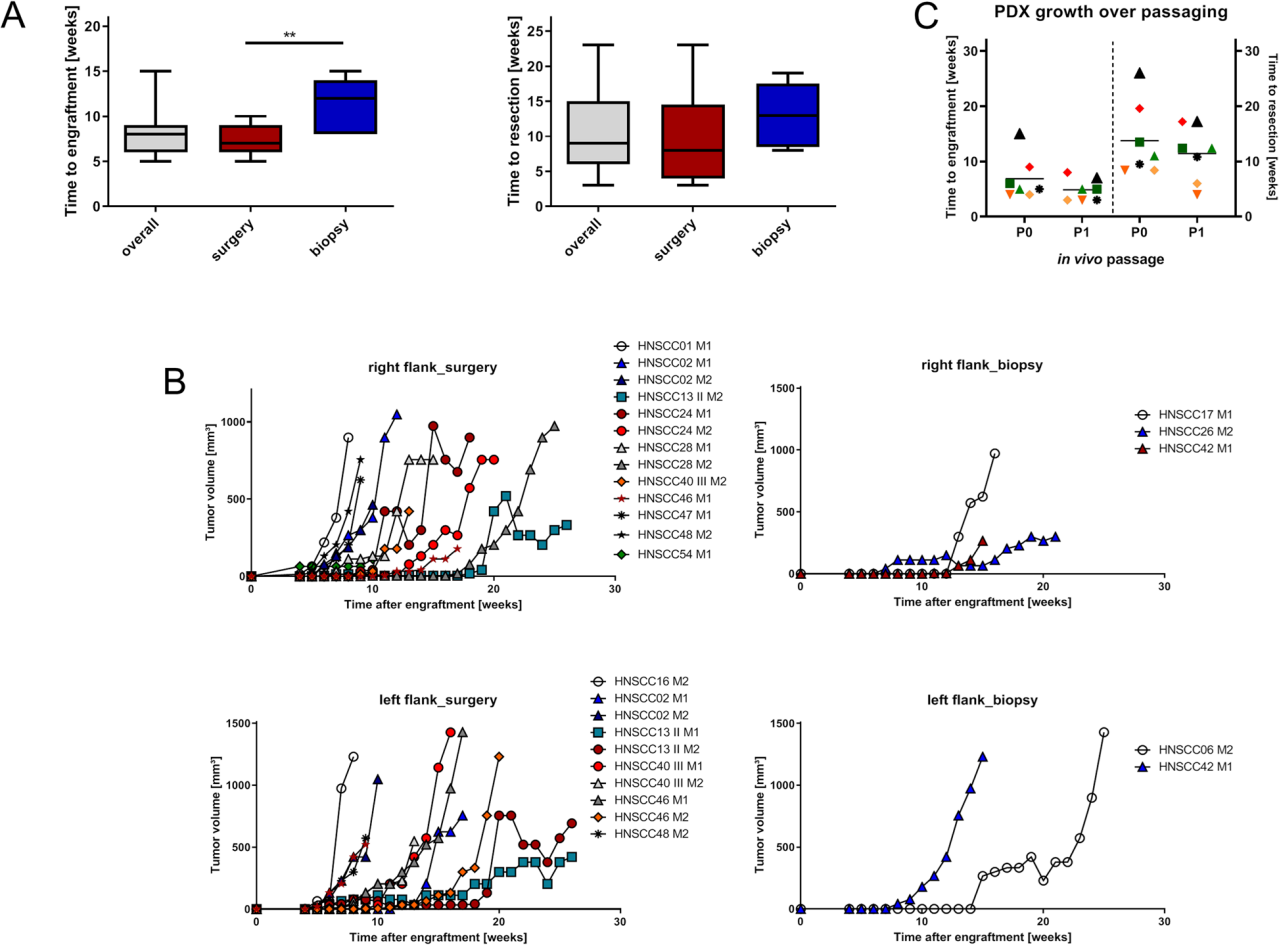
PDX formation, success rate, and growth kinetic in NSG mice. **A** Boxplots depicting the time from implantation to engraftment (appearance of palpable tumor in the flanks) and the time from the engraftment to resection when tumors reached maximum sizes [1500 mm^3^]. Whiskers show the minimum and maximum. **p < 0.01, unpaired t-test (two tailed). **B** Growth curves of the individual PDX: Each line represents a PDX grown in P0 in one mouse. M – mouse. **C** Growth acceleration after passaging: Scatterplot depicting the time from implantation to engraftment and resection for the initial implantation (= P0) and after cryopreservation and replantation into NSG mice (= P1). Each dot is representative for an individual HNSCC case using the same symbol and color for P0 and P1

Engraftment was obtained in 16 cases, yielding an overall efficacy of 36.4%. The engraftment rate was higher for surgical specimens (52.4 *vs*. 21.7%) and engraftment time was shorter (6.9 ± 2.4 *vs.* 10.6 ± 3.8 weeks; *p* < 0.05; Fig. 3A). After successful engraftment, PDX had comparable growth kinetics: Surgical specimens reached the maximum size in 9.5 ± 4.8 weeks and biopsies in 12.2 ± 4.6 weeks (Fig. 3A). Some aggressive cases grew in several flanks/implantation sites, while other cases did not engraft at all (Fig. 3B). Therefore, the positive flank related engraftment rate was 59.7% (Table 2). Again, the number of individual PDX/tumor was higher for surgical specimens (77.3%, Fig. 3B). Still, positive engraftment was seen in 37.5% of biopsy samples.

**Table 2:** Overview on engraftment efficacy in NSG mice comparatively shown for biopsies and surgical specimen

Analyzed parameter	N	%
PDX engraftment [total]	16/44	36.36
Biopsies	5/23	21.73
Surgical resection specimen	11/21	52.38
Positive flanks [total]	43/72	59.72^a^
Biopsies	9/24	37.50^a^
Surgical resection specimen	34/44	77.30^a^

^a^taken from a total of two mice/case each implanted with two tumor fragments (left and right flank)

Notably, four PDX were established from p16^+^/HPV-driven HNSCC cases (HNSCC06, HNSCC26, HNSCC42, and HNSCC54); three of them were taken from biopsies. Hence, the p16 status did not impact engraftment efficacy. Additional clinical parameters such as age, smoking, tumor localization, TNM stage and grade did not correlate with the engraftment. The same was true for the sampling related factors ischemia time and sample size (Table 3).

**Table 3:** Correlation analysis for PDX formation of HNSCC cases between biopsies and surgical specimen

Spearman correlation	*p* Value	Correlation coefficient^a^
Sample type	0.912	0.016
Sample size	0.300	-0.154
Age	0.887	0.021
Ischemia time	0.808	0.036
Ki-67 index	0.770	-0.043

^a^1 = perfect positive correlation; -1 = perfect negative correlation; 0 = no correlation

Serial passaging of the first seven established PDX models into P1 NSG mice was successful. All PDX grew after passaging and showed growth acceleration (time to tumor resection: 11.4 weeks vs. P0: 13.8 weeks) (Fig. 3C).

### Histomorphology is preserved in PDX

**Fig. 4:**
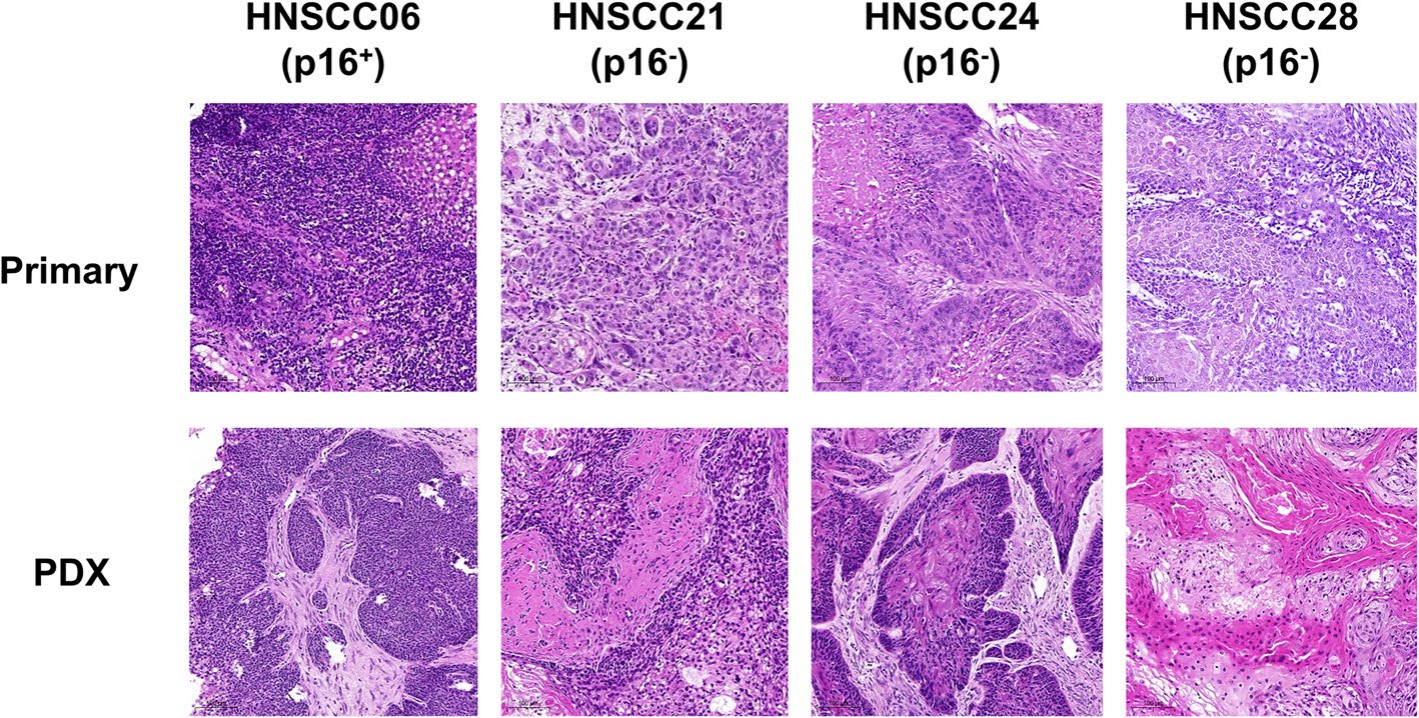
Histology of matched primary tumors and PDX models. HE histology represents maintenance of HNSCC tumor morphology following xenografting

**Fig. 5:**
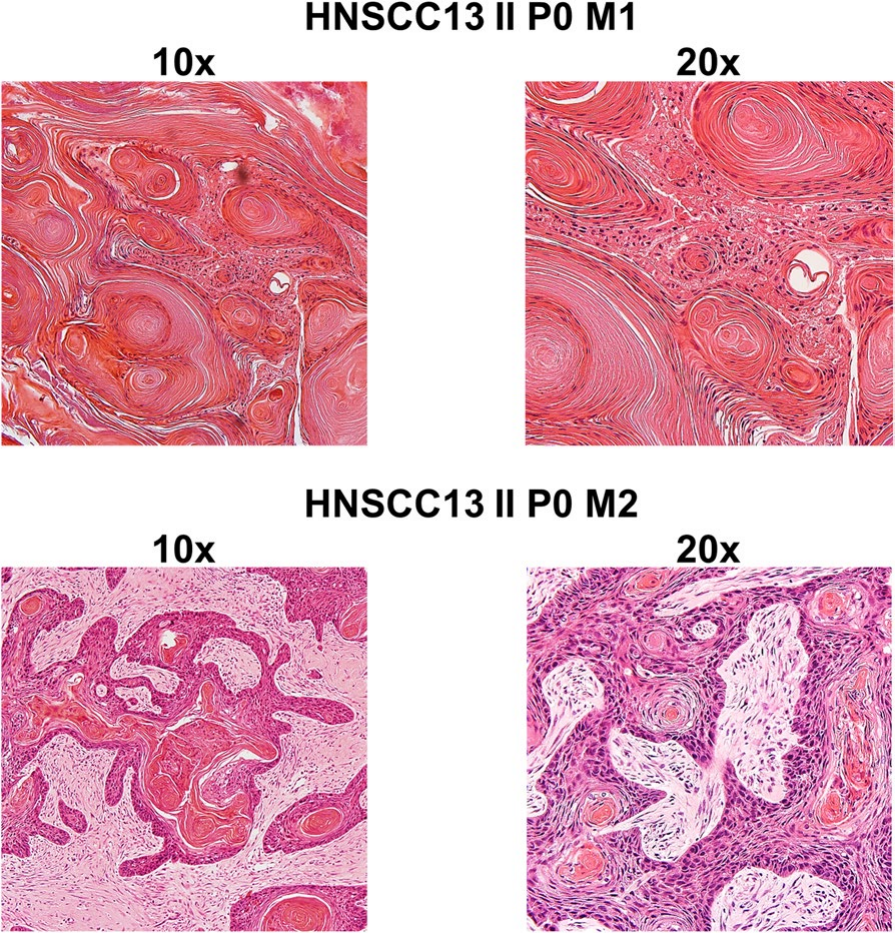
Intratumoral heterogeneity. HE staining of two individual PDX from case HNSCC13. Both PDX models preserve keratinization of the patient tumor. While HNSCC13 P0 M1 had strong keratinization, the corresponding PDX HNCCC13 P0 M2 had only few keratinizing areas

All PDX closely resembled their primaries (Fig. 4). Tumor architecture, growth pattern, cytological features and stromal architecture were principally preserved. Besides, tumor differentiation (i.e., poor, moderate, or well) largely matched between patients and PDX models (Fig. 4). The PDX reflected intratumoral and intertumoral heterogeneity. Sometimes there were minor differences in tumor morphology (degree of keratinization) between individual mice: HNSCC13 P0 M1 showed strong keratinization, whereas in HNSCC13 P0 M2 the number of keratinizing areas was moderate (Fig. 5).

Regarding the biopsies and surgical specimens, no differences in preservation of morphology could be detected.

### Tumor microenvironment of patient tumors and PDX

**Fig. 6:**
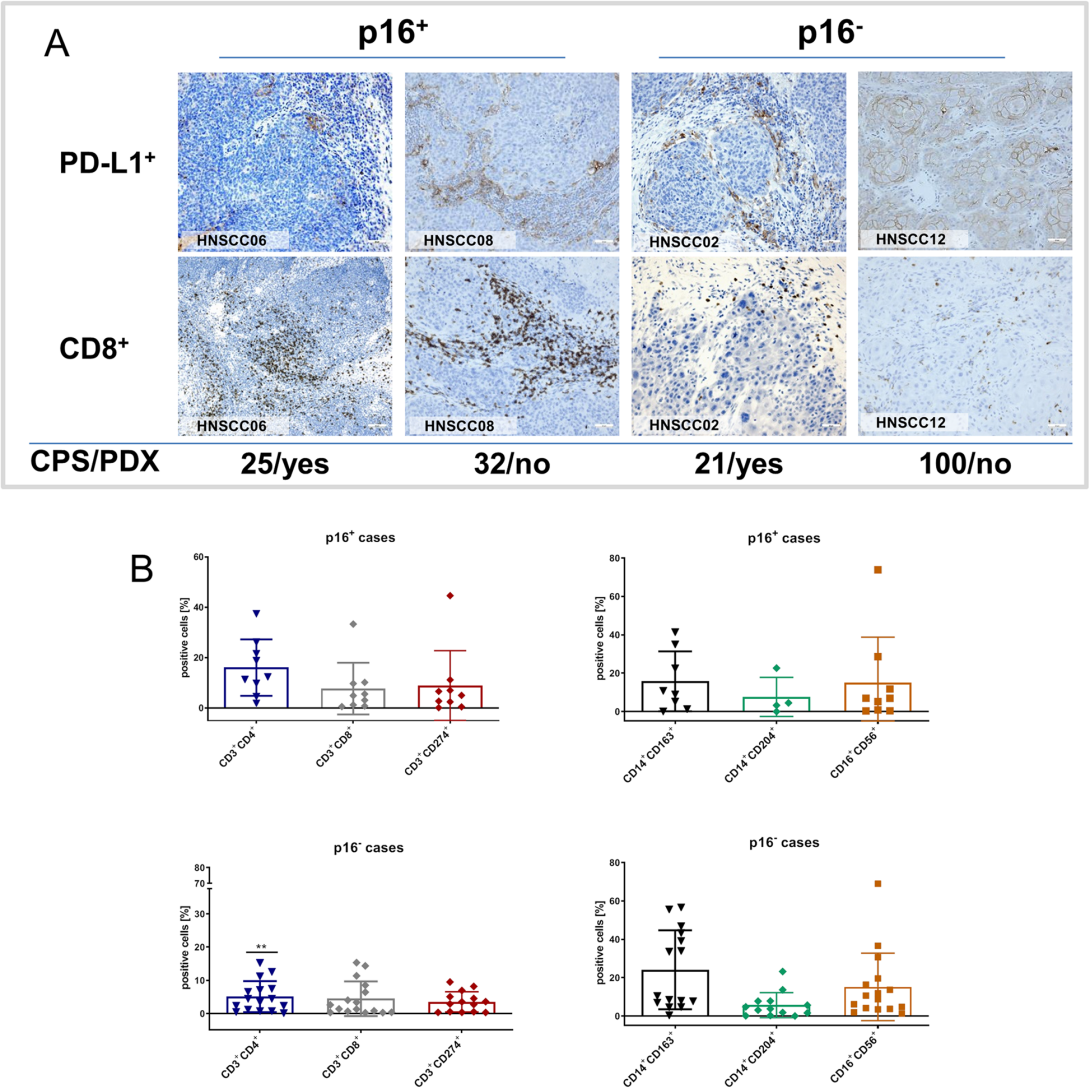
Tumor microenvironment phenotyping by flow cytometry and immunohistochemistry of p16^+^ and p16^−^ HNSCC. **A** Scatterplot depicting the percentage number of positive cells stained with the appropriate monoclonal antibodies followed by flow cytometric analysis and measuring 50,000 events in a live gate. Data analysis was performed using BD FACSuite software. HNSCC cases were separated according to the p16 status (i.e. p16^+^ of p16^−^). The number of tumor-infiltrating macrophages (CD14^+^CD163^+^ and CD14^+^CD204^+^) correlated with engraftment success. **p < 0.01 p16^+^ tumors vs. p16^−^ tumors; Mann Whitney *U* test. **B** Representative images showing PD-L1^+^ tumor and immune cells as well as infiltrating CD8^+^ cells in primary tumors. Tumor resection specimens were stained with the appropriate antibodies as stated in material & methods. The combined positive score (CPS) was calculated considering PD-L1 positivity in tumor and tumor-infiltrating immune cells. Additional information on engraftment success is given in the lower part (PDX – patient derived xenograft)

Immunohistochemistry and flow cytometry revealed correlations between PDX engraftment, PD-L1 expression, and macrophage infiltration (Fig. 6). Immunohistological quantification of PD-L1 using the combined positive score (PD-L1 positivity in tumor and tumor-infiltrating immune cells), showed a trend towards better engraftment for low combined positive score (CPS ≤ 10); whereas cases with high CPS were more likely to be rejected (CPS [yes] *vs*. CPS [no]: 18.22 vs. 33.41; Pearson r: -0.109] (Fig. 6A).

The leukocyte infiltration of the tumor microenvironment was quantified by flow cytometry. Twenty five cases were analyzed (p16^−^: 16 cases; p16^+^: 9 cases). Leukocytic infiltration was heterogeneous (Fig. 6B), but p16^+^ tumors showed a characteristic infiltration pattern. We identified high infiltration with CD3^+^CD4^+^ helper (*p* < 0.01 *vs*. p16^−^ tumors) and CD3^+^CD8^+^ cytotoxic T cells, along with elevated numbers of CD3^+^CD274^+^ T cells. CD14^+^CD163^+^ and CD14^+^CD204^+^ macrophages were less frequent in p16^+^ cases. CD16^+^CD56^+^ natural killer (NK) cells were heterogeneous irrespective of the p16 status.

Correlation of the innate immune cell compartment with PDX engraftment showed that engraftment was more frequent in cases with high numbers of CD14^+^CD163^+^ macrophages (Pearson r: -0.757; *p* < 0.05) and low numbers of CD14^+^CD204^+^ macrophages.

### Molecular pathology is preserved in PDX

**Fig. 7:**
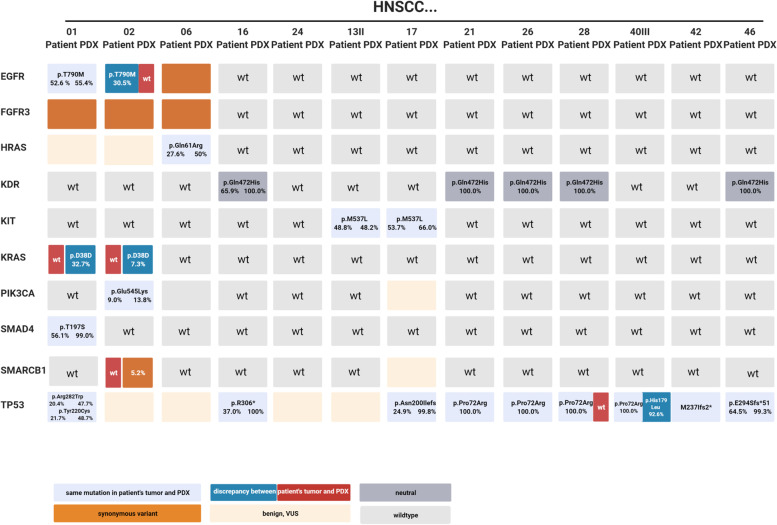
Molecular profile of matched primary tumors and PDX models. Nucleic acids were isolated from snap frozen samples and stored at -80 °C. Purified gDNA samples were than used to detect genomic alterations using the Illumina Cancer Hotspot Panel (Illumina, Berlin, Germany) covering mutations in 50 different genes with an iSeq100 sequencing system (Illumina) according to the manufacturer’s protocols. The specific mutations along with the variant allele frequency are depicted in the boxes. Differences between primary patients’ samples and matched PDX are highlighted. Mutations were classified in the following categories: non-synonymous—variant is given in the box (in case of the same mutation in the primary and PDX: light blue, difference between primary and PDX: red/blue); synonymous: orange; benign, VUS: light-orange; neutral: grey. The latter is defined as “passenger” mutation that does not play a role in HNSCC; wt – wildtype: light grey. Created with *biorender.com*

The mutational profile was studied in 13 patient samples and corresponding PDX. The overall number of genomic alterations in the cancer hotspot panel was low: *TP53* (61.5%) and *KDR* (38.4%) were the most affected genes. The direct comparison of the molecular fingerprint identified few discrepancies in most commonly affected genes (Fig. 7). In the PDX of HNSCC02, a *KRAS* mutation (c.114 T > C; variant allele frequency (VAF): 7.3%) and *SMARCB1* mutation (VAF: 5.2%) was detected. Vice versa*,* the patient tumor sample harbored an *EGFR* mutation (c.2361G > A; VAF: 30.5%) that was lost in the PDX. In HNSCC01, a *KRAS* mutation (c.114 T > C, VAF: 32.7%) was exclusively seen in the PDX. It can be assumed that intratumoral heterogeneity explains these findings best.

## Discussion

Patient-derived xenografts (PDX) maintain morphology and molecular profiling of the original tumors, thus providing a platform for the examination of disease biology, biomarkers, and novel therapeutic agents. However, restricted availability of tumour samples hindered the widespread use of PDX. In line with previous research on different cancer entities [7, 10, 27–29], we hypothesized that (pre-treatment) endoscopic biopsies contain sufficient viable tumor for PDX growth, in a clinically relevant time frame [21, 30].

Therefore, a comparative analysis on engraftment efficacy of tumor biopsies and surgical specimens was conducted. While both biopsy and surgery specimens were suitable for PDX formation in vivo*,* the engraftment rate was substantially lower for biopsies*.*

Recently, PDX engraftment from biopsies has been tested for many cancer entities. The success rates were 3 to 90% using fine- and core needle laparoscopic biopsies [15, 22, 23, 31, 32]. In line with our findings, direct comparisons with surgery sampling showed mainly lower biopsy engraftment rates. For HNSCC, Lilja-Fischer et al. implanted mainly HPV^+^ oropharyngeal cancer biopsies with a success rate of 33%, which is similar to our biopsy engraftment [24]. In conflict with these results, Kang et al. reported a superior HNSCC biopsy engraftment of 100% compared to 16% of surgery samples, yet in a very small patient cohort [25]. However, both groups did not specify whether the biopsies were diagnostic tonsillectomies, lymph node metastasis extirpations or endoscopic biopsies. Take rates approaching 100% are more typical for metastatic lymph node biopsies, which are not part of routine HNSCC diagnostics. Reviewing the present results in the scope of previous research, we expect engraftment rates of ~ 30% for biopsies and 50–70% for surgical specimens in HNSCC. However, comparisons of engraftment rates are impaired by limited standardization: Depending on the authors, the engraftment rate has been reported after one, two or three passages. More differences comprise the mouse strain (complex immunodeficient vs. athymic), the number of animals/tumor and the implantation technique (orthotopic vs. subcutaneous vs. kidney capsule vs. muscle). More standards are highly desirable to reduce laboratory animal expenditure.

A critical factor for engraftment efficacy is tissue ischemia and cryopreservation of tumor samples prior to engraftment that impair viability and thus PDX formation [33–37]. Tissue ischemia has been controlled by pathological diagnostics, processing, and viable freezing within 120 min. Immediate tissue transfer into mice is supposed to enable the highest yield of viable cells, but it also requires a complex infrastructure including timing of surgery, availability of laboratory personnel and accessibility to mice at a specific age (usually < 3 month). We therefore decided to cryopreserve fresh frozen tumor samples prior to engraftment and our engraftment efficacy was comparable to previous research using direct implantation. The standardized cryopreservation enables implantation of several tumor samples simultaneously and re-implantation of frozen backup samples if initial PDX formation fails. Freezing also facilitates reanimation of early passaged tumors at later time points. To date, all individual PDX cases were cryopreserved in P0 and seven have been replanted into NSG mice. All of them grew successfully in P1.

Additional to the higher engraftment rate, surgery samples showed an accelerated in vivo growth. After tumor formation, the growth kinetics was the same in both groups. Handling time, specimen size, p16 status, Ki-67 index, patients’ clinical characteristics and implantation (3 × 3 × 3 mm^3^ fragments) did not differ between the study groups. As sampling quality does not appear to be the primary reason, it can only be speculated on the underlying causes for impaired biopsy engraftment. One plausible explanation may be sampling of less proliferating tumor cells in biopsies because of intratumoral heterogeneity [32]. Likewise, the tumor microenvironment at the invasive margin may have played a role, too. Biopsies are usually taken from exophytic tumor areas to avoid bleeding and tumor cell dispersion: These areas are exposed to air, toxins and microorganisms. Also, basal proliferating areas remain untouched. In contrast, PDX of surgical specimens originate from central areas of the tumor to ensure safe pathological margin diagnostics. If the surgery samples are large and margins safe (e.g. laryngectomy) (I) viable cells from the cancer invasion front may be selected and (II) specific areas (center vs. margin) of the tumor may be punched to consider heterogeneity.

To improve engraftment rates, orthotopic implantation of HNSCC biopsy pieces might provide a good alternative. Also, metastasis and accurate mimicry of the original tumors’ environment can be anticipated. Another way to increase the number of PDX models from biopsies is a preselection of the tumor type (e.g. focusing only on highly malignant/aggressive cases) or ex vivo enrichment of tumor cells via tumor-surface antigen-based sorting/separation (such as EpCAM or cancer-initiating/stem cells), followed by co-implantation with non-malignant stromal cells. Finally, grafting of three-dimensional patient-derived tumor organoids or even circulating tumor cells is increasingly applied in preclinical research and may help to ameliorate success rates prospectively [38].

Even though surgery samples have many advantages, including non-resectable advanced cancer biopsy samples in preclinical models is crucial. Since these tumors are usually treated by radiation or chemotherapy, drug response prediction would be highly desirable. Additionally, 75% of the p16^+^ PDX originated from biopsies in this study. HPV-related HNSCC PDX underline the importance of biopsy xenografting in cancer entities, which are mostly treated by irradiation therapy. Improvement of biopsy engraftment could also enable repetitive PDX generation from pre-therapeutic endoscopy, surgery, post-radiation endoscopy and cancer recurrence to study cancer progression and clonal selection.

Previous studies described an association of clinical and pathological features between tumors with rapid and slow PDX growth. In pancreatic cancer, for instance, rapid growth was significantly associated with male gender and lymph node metastases [21]. Likewise, rapid PDX growth was associated with a poor outcome in melanoma and HNSCC patients [10, 30]. A recent study described an association of tumor mutational burden and reduced E7/p16^INK4A^ levels with p16^+^ HNSCC PDX and organoid engraftment [27]. Hence, successful and rapid PDX establishment is suspected to be predictive for increased risk of recurrence and poor outcome. Since the biobank has been established recently, survival rates could not be calculated. However, previous data on the correlation of PDX engraftment and patient outcome can be confirmed at least partially: One PDX was established from a chemoradiation refractory patient (HNSCC48) who deceased shortly after salvage surgery. This PDX grew rapidly and growth accelerated after serial passaging.

Another important factor in HNSCC research is the tumor microenvironment, since HNSCC naturally shows high immune cell infiltration. Fuji et al., described the impact of the tumor microenvironment on engraftment efficacy of colorectal cancer specimens and concluded that tumor-infiltrating lymphocytes can inhibit engraftment by exerting suppressive effects on tumor growth [39]. The tumor microenvironment was scanned with immunohistochemistry and flow cytometry in the present work. Both methods confirmed increased immune activity in p16^+^ tumors as part of the immune response to the viral infection compared to p16^−^ tumors. Regarding the influence of the TME on PDX growth, immunohistochemistry revealed that low CPS scores were associated with high engraftment rates. Flow cytometric TME analysis identified (tumor associated) CD14^+^CD163^+^ and CD14^+^CD204^+^ macrophages as critical determinants for PDX propagation. Both subtypes are strongly associated with the M2-like phenotype. In breast and lung cancer, circulating CD14^+^CD204^+^ cells are representative for an advanced tumor stage and contribute to metastasis [40, 41]. In esophageal cancer, the high infiltration of CD163^+^ macrophages is significantly associated with chemoresistance [42]. Because of the value of tumor microenvironment analysis for PDX engraftment and biomarker identification, flow cytometry could become a method of choice for quantitative examination of the immune infiltration. Flow cytometry is timesaving and measures multiple cell types simultaneously in small tumor samples (compared with immunohistochemistry). A major constraint of flow cytometric tumor microenvironment phenotyping is the lack of spatially resolved measurements regarding leukocyte localization within the tumor and its interaction with different cell types, such as tumor cells and cancer-associated fibroblasts. A very recent study integrated histomorphological patterns of immune cell infiltration and mRNA expression data of immune genes in HNSCC. In this study, the status of tumor-infiltrating lymphocytes in the intra-epithelial and stromal compartment was identified as non-redundant biomarker in HNSCC that should be evaluated separately [43]. In the present study, immunohistochemistry was combined with flow cytometry and it suggests the latter as an important add-on tool for tumor microenvironment analysis in preclinical models.

Finally, this work has several limitations. One major limitation is the partial molecular characterization using the 50 gene comprising Illumina Cancer Hotspot Panel. However, HNSCC is characterized by heterogeneous (epi-)genetic alterations and several comprehensive studies did not find reliable molecular biomarkers for PDX engraftment. As the focus was on the tumor microenvironment; applying flow cytometry and immunohistochemistry could identify macrophages differentiation and PD-L1 immune status as potential biomarkers for PDX engraftment. A second limitation relates to tumor heterogeneity: Implantation, histology and cancer sequencing were performed from one sample. Joining punches from different areas may preserve heterogeneity in future studies. Next, the PDX suffer from a selection bias in favor of advanced cancer. However, most HNSCC cancers are already advanced on diagnosis and most T1/2 lesions are sufficiently treated by transoral surgery. Therefore, advanced disease models are more valuable for preclinical research. Still, the results on implantation of specimens from small biopsies may also enhance research on PDX from early-stage lesions.

This study has implications for HNSCC PDX-based research: We have established and characterized new p16^+^ and p16^−^ in vitro and in vivo models from primary, recurrent and metastasized HNSCC. The new PDX represent morphology and molecular alterations of the patients’ tumors. Using endoscopic biopsies, this new PDX models comprise advanced disease patients that were not eligible for surgery. In this context, the correlation between PDX growth and clinical outcome could lead to the identification of clinical biomarkers.

Even though the engraftment rate is lower, PDX from biopsies are essential to include aggressive recurrent and metastasized carcinomas -that cannot be treated by surgery- in preclinical research. Since PDX engraftment from biopsies is feasible, routine implantation of endoscopic biopsies should be implemented in HNSCC research projects. As a result, biopsy PDX might play a crucial role in personalized therapy for HNSCC patients in advanced tumor stage.

## Conclusion

This study describes the successful establishment of patient-derived xenograft models from head and neck cancers obtained from endoscopic biopsies and surgery resection specimens. Additionally to the higher engraftment efficacy of the latter compared to the former, intratumoral M2-like macrophages as well as a low PD-L1 expression on tumor and immune cells were identified as independent positive predictors for engraftment. Vice versa, the p16 status had no impact on engraftment efficacy. Finally, PDX models from both sources were successfully transferred and expanded thus broadening the resources for preclinical drug response analyses.

## Supplementary Information

**Additional file 1: Supplementary Table 1**. Patient characteristics and clinical information

## Data Availability

All data generated or analyzed during this study are included in this published article [and its supplementary information files].

## Abbreviations

CUP: Cancer of unknown primary
HNSCC: Head and neck squamous cell carcinoma
HPV: Human papilloma virus
PDX: Patient-derived xenografts
VAF: Variant allele frequency
